# Over-Application and Interviewing in the 2021 United States Primary Care Virtual Recruitment Season

**DOI:** 10.7759/cureus.39084

**Published:** 2023-05-16

**Authors:** Ephy R Love, Jason I Reminick, Franklin Dexter, Risë Goldstein, Brett Robbins, Suzanne B Karan

**Affiliations:** 1 Thalamus, SJ Medconnect, Inc., Santa Clara, USA; 2 Anesthesia, University of Iowa, Iowa City, USA; 3 Research, National Resident Matching Program, Washington DC, USA; 4 Internal Medicine - Pediatrics, University of Rochester, Rochester, USA; 5 Anesthesiology, University of Rochester, Rochester, USA

**Keywords:** data driven policy, pediatrics, family medicine, internal medicine, primary care, residency interviews, over-interviewing, residency, medical education, gme

## Abstract

Importance

Over-application and interviewing are believed to be widespread in residency recruitment. These may have increased during the 2021 virtual recruitment season. The increase does not correspond to an increase in available residency positions and likely results in more interviews with low probabilities of yielding matches. Prior work demonstrates that such marginal interviews are identifiable ‑ from key explanatory factors like same-state for interviewee and program ‑ in sufficient volume to allow programs to substantially decrease interviews.

Objective

To evaluate the importance of same-state relationships in primary care and to determine the extent of over-interviewing in the 2021 virtual recruitment season.

Design

The National Resident Matching Program and Thalamus merged match (outcomes) and interview (explanatory variables) data from primary care specialties (family medicine, internal medicine, pediatrics). Data were analyzed by logistic regression, trained on the 2017-2020 seasons, and projected on the 2021 season for testing.

Setting

The setting was the 2017-2021 main residency matches.

Participants

This comprised 4,442 interviewees applying to 167 residency programs in primary care.

Intervention

This included the transition to virtual recruitment from in-person recruitment in the 2021 residency recruitment season.

Measurements

A total of 20,415 interviews and 20,791 preferred programs with program and interviewee characteristics and match outcomes were included.

Results

Same-state geographic relations predicted match probability in primary care residency interviews better than medical school/residency affiliation, with 86.0% of interviewees matching consistently with their preferences for the same state. Same-state was more effective than medical school affiliations with programs in predicting matching. Eliminating interviews with less than a 5% probability of matching (upper 95% prediction limit) removed 31.5% of interviews.

Conclusions and relevance

The large number of low-match probability interviews demonstrates over-interviewing in primary care. We suggest that programs eliminate interview offers to applications falling below their chosen match probability threshold.

## Introduction

Residency recruitment is a historically challenging marketplace that has been further complicated by the coronavirus disease 2019 (COVID-19) pandemic. Recent adjustments faced by both applicants and programs include a pivot to virtual interviewing, decreased availability of “away rotations,” and conversion of the US Medical Licensing Examination (USMLE) Step 1 score to pass/fail. Application costs vary by specialty but may exceed $5,000 per applicant [1-2]. Though travel time has decreased, both the cost of travel and application fees have increased, as have the number of application submissions per applicant [3]. Primary care specialties ­­- internal medicine, family medicine, and pediatrics - include the majority of the roughly 50,000 annual residency applicants, comprising ~40% (~20K), ~20% (~10K), and ~10% (~5K), respectively [3]. The time required annually for programs to filter through the resulting pools of 2-2.5M total primary care applications, particularly with the recent heightened emphasis on holistic review, is daunting. With application inflation and over-interviewing (interviewing despite the low probability of match), concerns over the sustainability of the resident recruitment process have prompted recent analyses by us and others of current efforts [4-6] and novel pilot proposals to ameliorate the situation [7-9].

The National Resident Matching Program (NRMP) and Thalamus (SJ MedConnect, Inc dba Thalamus, Santa Clara, CA; https://thalamusgme.com) collaborated to combine interview with match data and examine the key variables driving the conversion of an interview to a match. Thalamus is a cloud-based graduate medical education interview scheduling software and management platform. Authors EL, JR, and SK are shareholders. This study extends previous work in anesthesiology [9-10] to the three primary care specialties (internal medicine, family medicine, and pediatrics), with a larger sample of interviews and matches. The current study sought to quantify the amount of over-interviewing in primary care and make recommendations for future interventions.

## Materials and methods

Replicating the methods from our two prior studies [9-10], we modeled the probabilities of interviews resulting in matches for the three primary care residencies. Please refer to Supplemental Section A.

Variables included and data sources

NRMP data included an applicant’s matched program (if matched), “preferred program” (the first ranked program on an interviewee’s rank order list), and rank order list length (the total number of programs submitted by each interviewee for the NRMP Main Residency Match). Accreditation Council for Graduate Medical Education identifiers were used for programs and Association of American Medical Colleges identifiers were used for interviewees. Data on interviews, as well as program and interviewee characteristics, were procured from Thalamus’ interview management platform and its application screening and review software, Cortex. All applicants and programs in the Thalamus database were successfully linked to programs and interviewees in the NRMP Match data. Note that an “applicant” to the match by definition attended at least one interview; therefore, the population of match applicants or “interviewees” does not include those who applied to residencies but received no interviews.

Data analyses

As in our prior work [9-10], data joining and preprocessing were performed by Thalamus, and then deidentified data were analyzed. The statistical study of these data was reviewed by the University of Iowa Institutional Review Board (IRB, 202103630) and determined not to be human subject research. Data sources and preprocessing are detailed in Supplemental Section A. Analyses were performed in R 4.0 statistical software; detailed code examples and a list of libraries used can be found in our prior work [10-11]. We compared the estimated parameters for the new primary care model based on data from 2017-2020, to the findings in prior work [10-11]. Next, we applied the trained model to 2021 data to identify interviews with low probabilities of resulting in matches and observe our cumulative and program-wise error rates and interviews reduced.

Our analyses addressed two objectives: first (inferential) - replication of the finding from our prior work [10-11], that same-state (the interviewee’s current address is in the same state as the interviewing program) is an important marker of match probability; and second (predictive) - modeling primary care match probabilities with respect to interview characteristics.

The same-state analysis required complete interviewee state, program state, and match/preference data, available from n1=30,211 interviewees and 1,479 programs.

Because of differences among residency markets in the three primary care specialties, we included a fixed effect for specialty. We found that the number of interviews per program divided by the number of matched residents varied considerably in 2021 among specialties, with markedly more interviews per matched position per specialty than in anesthesiology (see details in Supplemental Section B). Because each interviewee can match at only one program, there will be substantially more over-interviewing in primary care than in anesthesiology.

For our second analysis, we computed program characteristics, such as average USMLE Step 1 and Step 2CK board scores for program residents (matched interviewees). We removed programs with fewer than five matched residents over five years (at most one per year) with complete characteristic data, to avoid mischaracterizing a program based on an inadequate sample. After we joined and limited the data, our sample comprised n2=20,415 interviews from 4,442 interviewees and 167 programs with all data available and meeting the above criteria. The data were split chronologically into train (2017-2020)/test (2021) sets (Table 1). The coverage of primary care programs in the qualified sample grew considerably, from 78 to 166 programs between 2017 and 2021 (Note: These are programs with complete features, after filtering described in Supplemental Section A). Because our study treated the 2021 season as the test set, the change in program-set size was not concerning with respect to temporal effects, as the new programs in the test set acted as an additional robustness test of model fit. Using the 2021 season as a test had the added benefit of checking robustness due to the COVID-19 pandemic and resultant virtual interviewing.

**Table 1:** Train (2017-2020) and test (2021) sample sizes of programs, interviewees, and interviews Each sample is stratified by specialty (family medicine, internal medicine, pediatrics).

	Match Seasons	Programs	Interviewees	Interviews
Train	2017-2020	78 (18,33,27)	2,226 (148,1013,1065)	6,962 (242,2696,4024)
Test	2021	167 (46,78,43)	2,216 (293,1240,583)	13,453 (1269,8518,3666)

Geographic investigation

**Figure 1:**
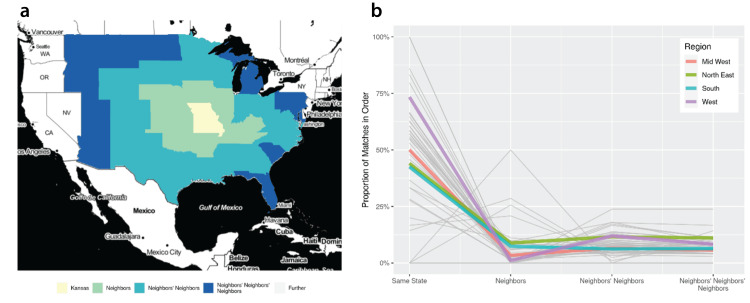
a) Shows a neighborhood ordering for the state of Missouri, immediate neighbors, neighbors’ neighbors, and neighbors’ neighbors’ neighbors. Missouri is shown as an example due to its centrality to the continental US and consequent lasting, clear visual pattern in its neighbors. b) Shows the rate of matching for the same state, neighboring states, neighbors’ neighbors, and neighbors’ neighbors’ neighbors. The light gray lines each represent a single state. The colored bold lines show trends aggregated by region. For all regions, the observed percentages of interviews resulting in a match drops as geographic distance increases between an interviewee and a program. This figure contains unadjusted percentages obtained from 20,132 interviews.

As in our prior work [10-11], for our primary outcome, same-state matching, we examined the percentage of interviewees who listed a same-state program as most preferred. Second, to study whether same-state preference was weaker or stronger than an applicant preferencing their home institution, we compared same-state preference and match rates to preference and match rates of programs affiliated with the interviewees’ medical schools. Then, third, to elucidate the impact of state boundaries, we measured the probability of applicants in each state matching within the state, within a neighboring state, or a state even further removed from their home state. We then used US regions defined by the US Census Bureau to test the probability of matching by US region. To visualize these data, state-wise trends by the US Census Bureau region were aggregated to provide an overlayed regional visualization of the effect of state distance on match probability by region. (See our prior study for a crosswalk of US states and regions [8]). In Figure 1, we plotted all states’ trends and regional trends, to depict the contribution of same-state geographic relation to match probabilities.

Logistic regression

The primary outcome of this paper was a logistic regression model predicting a binary (match) outcome by interview characteristics, fit to pre-virtual interviewing data. The model simultaneously estimates the contributions of variables, including programs’ and interviewees’ characteristics, to the probability of each interview resulting in a match. We provide descriptive statistics for regression variables in Supplemental Sections C and D. Predictions from the logistic regression are probabilities of an interview resulting in a match. Using corresponding standard errors in the logit scale, we computed an upper prediction limit for each probability (a measurably conservative estimate of match probability).

To make them useful to a training program, the resulting probabilities must be categorized according to some strategy for a program to reduce interviews (i.e. should an interview be taken when there is a <5% chance of matching or less than 1%? And how conservative should we be in our estimates considering the variance between interviews within a program?). Additionally, a program should use the upper prediction interval limit, rather than the estimate of the probability, to account for variation in the prediction. Each program must therefore choose its match probability threshold, beneath which interviewees are not considered, as well as an upper prediction interval limit. We offer several reasonable thresholds (1%, 5%, and 10%) with varying upper prediction interval limits (90%, 95%, and 99%) to provide examples of how to determine a threshold that makes sense for the individual program’s characteristics and risk tolerance (how a program should weigh the risk and cost of a change in the rank list against the cost of conducting an interview is not analyzed in this work and is left to the program to determine).

Ethics statement

Interviewee and residency program data were gathered from five sources (Thalamus, NRMP, Doximity, ACGME, and U.S. News and World Report). Those data were merged and de-identified. The University of Iowa Institutional Review Board (IRB, 202103630) determined that the study of this de-identified data does not meet the regulatory definition of human subjects research.

## Results

From 2021 interview and match data, internal medicine, family medicine, and pediatric residency programs interviewed an average of 32, 41, and 32 applicants per matched position, respectively, despite only 7.5%, 4.3%, and 1.8% of positions, respectively, going unfilled in the 2020 Main Residency Match prior to the Supplemental Offer and Acceptance Program (SOAP). The 2020 Main Residency Match represented the largest NRMP match in history up to that year for each specialty and saw an overall position fill rate of 95.2%.

Our primary, inferential analysis of same-state matching showed an overall same-state concordance of 85.9% with 9,714 (32.2%) interviewees both preferring and matching in a residency program within the same state as their medical school and 16,223 (53.7%) interviewees both preferring and matching into a program in a different state. Among those who matched in a different state, 1,915 (6.3%) interviewees preferred same-state (ranked a program in the same state first), but matched out-of-state. A total of 2,359 (7.8%) interviewees ranked programs first preferred out of state but matched in the same state. These results indicate that 83.5% of interviewees who wanted to match in the same state did so. Conversely, Alpha Omega Alpha (AOA, the national honor society of allopathic medicine) membership is a sought-after applicant characteristic, but only 35.2% of interviews involved either programs less than half of whose residents were, and interviewees who were not, AOA members, or programs more than half of whose members were, and interviewees who were, AOA members. Its match-predicting accuracy was only 35.2% (i.e., a third of matches can be accurately predicted by the interviewee (program)/AOA status (average) concordance; see Supplemental Section D). Same medical school program affiliation had match-predicting accuracy comparable to same-state, at 90.4%, but fewer students (14.5%) preferred an affiliated program. Specifically, 3,491 (11.6%) of interviewees preferred and matched an affiliated program (See Note *). Consequently, same-state predicted 178.3% more matches than program-medical school affiliation.

We also tested the relative efficacy of geographic relationships related to same-state, specifically neighbors (i.e., a bordering state), neighbors’ neighbors (i.e., a bordering state to a bordering state), and neighbors’ neighbors’ neighbors (i.e., a bordering state to a bordering state to a bordering state). Figure 1 shows the percentage of matches that came from each neighboring state grouping when comparing the interviewees’ current address to the residency location. Figure 1 shows a steep drop-off moving from the same state to a neighboring state, demonstrating the value of same-state as compared to a regional association. The finding that a combination of same-state and other interview characteristics (e.g. AOA, Doximity rank) correlated with match probability was confirmed by logistic regression (Table 2), as well.

**Table 2:** Summary of logistic regression fit to data from 2017-2020 using 6,962 observations The variables are defined as follows: a) *sameState* is binary classification that indicate whether an interviewee was currently living in the same state as the program. b) *usmleAveD* (USMLE average difference) takes the interviewee’s average USMLE 1,2CK scores and subtracts them from the programs historic mean average score of matched residents. c) *usnwrAveD* (USNWR average difference) takes the interviewee’s medical school USNWR research ranking and subtracts it from the programs historic mean of matched residents. d-f) *progregion* (*North East, Mid-West, South, West*) (Program region) is the program’s USCB Region. These are categorical variables that are included in regression by encoding into 0/1 binaries with names concatenated (typical R usage). g) *intCount* (Interview Count) is the count of interviews recorded in the data for the interviewee. h)* Doximity Rank *is the Doximity ranking of the program (lower is better). i-k) *aoaCat* (*tt,tf,ft,ff*) (AOA Category) is a categorization where a program is AOA “true” if that program is above the median of all programs in terms of percentage of interviewees who are AOA “true”. These variables as well are included as 0/1 binaries with concatenated names. See Supplemental Section D for full details on variable coding. l,m) *spec* (*Internal Medicine, Family Medicine, Pediatrics*) (specialty) is a categorization determined by the interviewing program’s specialty. ^1^Coefficients reported in logit scale, as estimated by the linear statistical model. ^2^Transformation of the parameter estimates in logit scale to odds ratio, and 95% two-sided confidence interval for the transformed parameter estimates.

	Variable	Coefficient^1^	Std error	P-value	Odds ratio (2.5-97.5% CI)^2^
a	Same State	0.75333	0.06748	<0.00001	2.124 (1.861-2.425)
b	Diff From Ave USMLE	0.02196	0.00251	<0.00001	1.022 (1.017-1.027)
c	Diff From Ave USNWR	-0.00582	0.00083	<0.00001	0.994 (0.993-0.996)
d	Northeast Program	0.06505	0.08698	0.45453	1.067 (0.900-1.265)
e	Southern Program	0.17191	0.09140	0.05999	1.188 (0.993-1.421)
f	Western Program	-0.06412	0.08225	0.43565	0.938 (0.798-1.102)
g	Interview Count	-0.39330	0.01877	<0.00001	0.675 (0.650-0.700)
h	Doximity Rank	0.00109	0.00028	0.00009	1.001 (1.001-1.002)
i	AOA (prog F, interv T)	-13.19723	155.54047	0.93238	0.000 (0.000-0.001)
j	AOA (prog T, interv F)	0.96140	0.09667	<0.00001	2.615 (2.168-3.168)
k	AOA (prog T, interv T)	0.92619	0.11847	<0.00001	2.525 (2.004-3.189)
l	Internal Medicine	-0.70809	0.15477	<0.00001	0.493 (0.363-0.666)
m	Pediatrics	-0.49795	0.15368	0.00119	0.608 (0.449-0.820)
	Intercept	0.31633	0.17767	0.07501	1.372 (0.969-1.945)

Reductions in interviews

Our second, predictive analysis showed that across primary care specialties in 2021, US Senior MD and DO interviewees submitted rank order lists totaling 122,146 cumulative ranks, with the qualifying study sample accounting for 13,170 interviews. Each rank represents an interview. Table 3 demonstrates that 7.6% of interviews could be eliminated with the median program experiencing only one change to its match results (using highly conservative 99% prediction limits and <1% thresholds). When using the moderately conservative 95% prediction limit and 5% match thresholds, interviews could be culled by 31.5%, eliminating 4,238 interviews annually with the median program experiencing only two changes to its final match list.

**Table 3:** Results of reducing interviews whose upper limit of probability of matching falls below a threshold at a one-sided upper prediction limit *False negatives are instances where an interview was recommended against, and the interview would have resulted in a match. The different rows of the table show results for several reasonable thresholds (1%, 5%, and 10%) with varying upper prediction interval limits (90%, 95%, and 99%) to provide examples of how to determine a threshold that makes sense for the individual program’s characteristics and risk tolerance.

Prediction limit	Threshold	Median program false negatives*	Total interviews recommended against (Internal Medicine, Pediatrics, Family Medicine)	Percent of interviews recommended against (Internal Medicine, Pediatrics, Family Medicine)
99%	<1%	1 (1,1,0)	1023 (829,183,11)	7.6% (10%,5%,1%)
99%	<5%	2 (1,2,2)	3963 (2913,934,116)	29.5% (34%,25%,9%)
99%	<10%	3 (1,2,1)	6375 (4559,1589,227)	47.4% (53%,43%,18%)
95%	<1%	1 (1,1,1)	1180 (954,206,20)	8.8% (11%,6%,2%)
95%	<5%	2 (1,2,2)	4238 (3088,1016,134)	31.5% (36%,28%,11%)
95%	<10%	3 (1,2,1)	6667 (4746,1658,263)	49.6% (56%,45%,21%)
90%	<1%	1 (1,2,1)	1245 (1002,218,25)	9.3% (12%,6%,2%)
90%	<5%	2 (1,2,2)	4354 (3178,1029,147)	32.4% (37%,28%,12%)
90%	<10%	3 (1,2,1)	6803 (4827,1693,283)	50.6% (57%,46%,22%)

**Figure 2:**
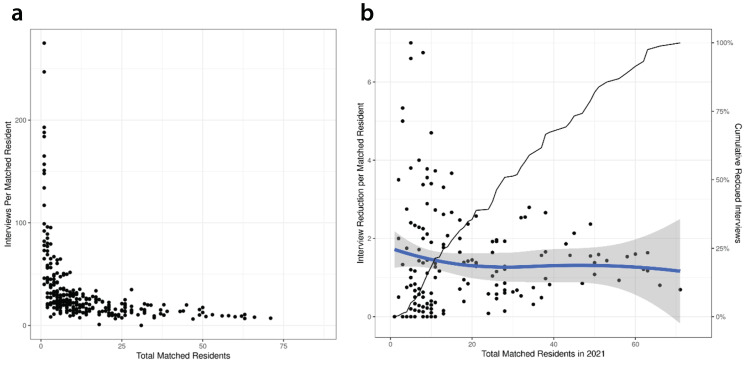
a) Program interviews per matched resident by total matched residents in the 2021 Match season. b) Interview reduction per matched resident for programs by the size of the program. Each point gives a program’s sum of reduced interviews per matched position (removing interviews with <5% chance of matching taking the 95% upper prediction limit) on the y-coordinate and the number of positions filled in 2021 on the x-coordinate. The black line shows the roughly linear, cumulative interview reduction as we include more programs from smallest to largest. The blue line gives a smoothed (LOESS with span=1) fit of the data, showing the over-interviewing cannot be attributed reliably to small versus large programs. LOESS: locally estimated scatter-plot smoothing

In an ancillary analysis, we found that smaller programs tended to interview many more interviewees per position than larger programs (Figure 2). The smaller programs were rural family medicine programs with a single position available annually, and the largest program was an internal medicine program with over 60 residency training positions available annually. Though small programs interviewed many more applicants per available position than large programs, over-interviewing was spread relatively homogeneously across programs in primary care and geographic regions.

Note*

Discordances were 2,033 (6.7%) interviewees not preferring an affiliated program and matching at the program, and 882 (2.9%) preferring an affiliated program and not matching at the program. An affiliated program connotes that the medical school and the residency share an institutional affiliation.

## Discussion

Together, our primary (inferential) and secondary (predictive) findings demonstrate substantial over-interviewing in the primary care residency market during the 2021 cycle with virtual interviewing. Though there have been calls to reduce applications and interviews [12], few evidence-based strategies are currently available [6]. Applying the results of our analyses to the 122,146 cumulative ranks submitted by US Senior MD and DO interviewees in primary care in 2021 (assuming a rank per interview, which may underestimate total interviews), suggests that 38,476 interviews could be eliminated annually.

Reducing application volume has substantial financial implications. Our model predicts that 31.5% of interviews in primary care specialties could be eliminated, with only minimal changes to final match lists. As an example, in 2020, 25,885 applicants to internal medicine submitted an average of 71 applications per person, totaling 1,837,835 applications (https://www.aamc.org/data-reports/interactive-data/eras-statistics-data). For primary care specialties, including internal medicine, applicants use Electronic Residency Application Service (ERAS), which charges fees per application that increase with the number of applications ($99 for the first 10 applications, 11-20 applications $19 each, 21-30 applications $23 each, and 31 or more applications, $26 each). Eliminating 31.5% of applications (22 applications/applicant on average) at the $26 threshold would remove approximately $14 million in application fees annually. While it is not possible to estimate the extent to which a decrease in interviews would contribute to a decrease in applications (as some applicants may still apply to more programs despite less chances of receiving an interview), a meaningful portion of these 14 million dollars would be saved. Beyond application costs, conducting interviews is an expensive, time-consuming process for programs, for whom reductions would also result in tens of millions of dollars in savings; further, it is a tremendously anxiety-provoking period for applicants [13-14].

We found that the same-state geographic relationship was an important indicator of match probability in primary care and more useful than medical school and residency program affiliation. Reassuringly for applicants, a large proportion (84%) of interviewees who wanted to match same-state did so. Our data showed that primary care programs interview many more applicants per position than specialty programs [10]. The potential benefit and interview burden reduction are thus greater for the primary care specialties. This study shows that the probability of an interview resulting in a matched resident can be modeled with a small set of characteristics: licensing exam score, medical school rank, and AOA status; and program state and Doximity ranking. This model could be extended with more explanatory variables for increased precision. For the analyses reported herein, this sparse model was chosen for its utility for residency programs and in order to compare (extend) to prior work.

This work has two main limitations: it does not include international medical graduates (IMGs) and does not incorporate data to identify underrepresented groups. To assess same-state geographic effects, this study, by design, had to exclude IMGs who live outside the US, despite the fact that they account for ~ 32.5% of filled positions in 2021 (https://www.nrmp.org/wp-content/uploads/2021/08/MRM-Results_and-Data_2021.pdf). This work should also be extended to assess variation in outcomes among underrepresented in medicine groups and to determine other predictors of matching for applicants and programs. Recent research has highlighted the importance of the UME to GME transition in diversifying the physician workforce [15].

Primary care residencies exhibit vastly greater variation in size among programs within specialties than what we observed in a prior study in a different specialty (anesthesiology). Small programs are compelled to interview many more candidates per position than large programs to reduce the risks of not filling. Therefore, their probabilities of matching each interviewee are substantially lower than in large programs. Smaller programs also tend to be in less urban geographies with smaller populations. We found that interview reductions driven by the implementation of this algorithm would follow program size in a roughly linear relationship (Figure 2). Thus, we suggest that policy considerations should not target specific programs interviewing many more than others. Instead, interview reductions using the algorithm presented in this work would best be applied to all programs in a specialty. Importantly, however, these results should not be interpreted to suggest that programs begin blindly culling interviews without an additional holistic review and an emphasis on recruiting a diverse applicant pool. Accordingly, this work can be subsequently extended to include parameters regarding diversity, equity, inclusion, and belonging.

## Conclusions

The transition from medical school to residency is a major source of anxiety for students. We identified large numbers of interviews with a very low likelihood of yielding matches in primary care during the 2021 cycle with virtual interviewing. Further, interviewing is only measured after programs have already filtered applications, implying additional over-application. This is the first study to quantify the number of interviews of marginal utility, implying that tens of thousands of interviews, hundreds of thousands of applications, and tens to hundreds of millions of dollars spent on residency application and recruitment are wasted. The residency application and interview markets require broad reform, which should be anchored in the best available evidence. These reforms will affect where physicians train and practice and will therefore have lasting impacts on American healthcare.

## Figures and Tables

**Figure 3:**
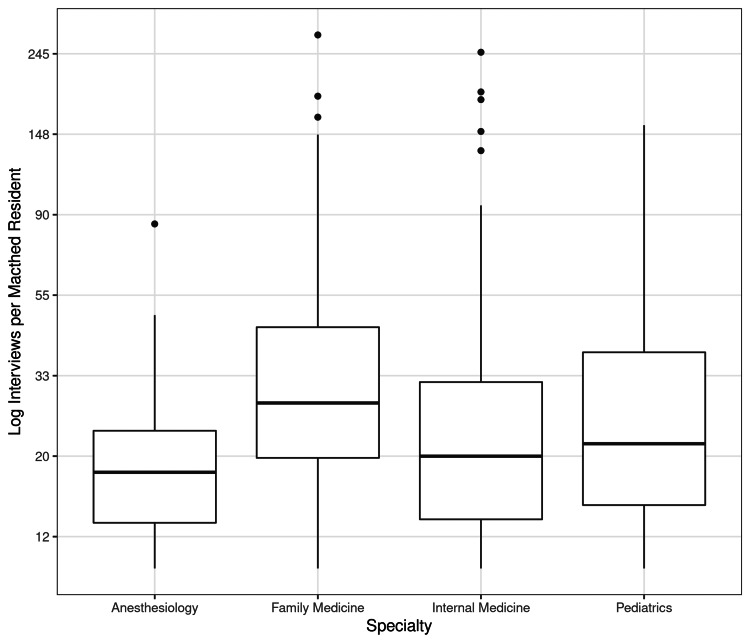
boxplot of logged interviews per matched resident for programs in the Thalamus interview sample (n=418 programs).

## Appendices

Supplemental content

A. Data details and preprocessing

Interviewee and residency program data were gathered from five software platforms (Thalamus, NRMP, Doximity, ACGME, and U.S. News and World Report). Those data were merged and de-identified. The University of Iowa Institutional Review Board (IRB, 202103630) determined that the study of this de-identified data does not meet the regulatory definition of human subjects research. Thalamus is a cloud-based, graduate medical education interview scheduling software and management platform (SJ MedConnect, Inc. dba ThalamusGME, Santa Clara, CA; https://thalamusgme.com/). Authors EL, JR, and SK are Thalamus shareholders.

Preprocessing by NRMP and Thalamus

Interviewee data was collected from applicants to primary care residency programs who scheduled their interviews via Thalamus. Program data were collected from the Accreditation Council for Graduate Medical Education (ACGME), Doximity^©^, U.S. News and World Report (USNWR©), NRMP, and Thalamus.

Thalamus’s data began with 58,299 interviewees and 70,983 interviews stemming from 310 programs in 42 states. NRMP data covered the population of 63,795 match applicants (presumably all of whom are interviewed at the programs to which they had applied), from 1,270 programs.

The first analysis in this work, comparing preferred and matched programs, required a current address state for the interviewee, medical school, and the interviewee had to match. Additionally, in order to test medical school affiliation, the medical school had to be listed as affiliated with at least one ACGME-accredited residency program. After merging data, limiting to complete cases, and deidentifying the records, this dataset included n1=30,211 interviewees and 1,270 matched primary care programs.

The second analysis in this work, predicting match outcomes from interview features, required many more data fields. The data used for each interviewee included medical school, current address-state, United States Medical Licensing Exam Step 1 and 2CK scores, Alpha Omega Alpha status, and Thalamus interview count. The datum of “current address-state” refers to the US state (and the District of Columbia) of the current address at which an interviewee resides at the time of application submission to the Electronic Residency Application Service.

Program-specific means were calculated using the data from the interviewees. The USNWR^©^ 2020 Medical School Rankings Research score ranks medical schools and assigns a numerical value for each rank, rank 1 being the highest-ranked school, etc. As a proxy for medical school prestige, the mean of those scores was calculated for each program among the interviewees who matched at each of the 310 programs. The address of the residency program used was that listed within the ACGME online Accreditation Data System (ADS). After calculating program-specific means, limiting to programs with more than five residents with complete data, and limiting interviewees to those with complete data, the dataset included n2=20,132 interviews from 4,024 interviewees and 166 programs.

Each interviewee was only interviewed during a single recruitment year for a postgraduate year (PGY)-1 or PGY-2 position. Interviewees have a single user account in Thalamus as defined by their American Association of Medical College identification (ID) number. It is a unique account defined by a unique number. The database query can search across interview seasons. No interviewees studied had interview invitations that extended across interview seasons. The number of PGY2 matches is smaller and therefore challenging to compare to a PGY1 position. Programs may also interview PGY2s differently and/or assess them differently (given they have intern year as an additional metric and number of data points by which to assess the candidate). All interviewees were successfully merged into their NRMP records. Identifying program features were removed from the dataset. The AAMC ID number and any other disclosing personal information of interviewees were removed.

Statistical Analyses of De-identified Data for Current Study

R version 3.6.3 (2020-02-29) and several open-source R libraries were used to complete analyses for the paper’s results. For the complete list of libraries used, see Supplemental Section B - Appendix of R Packages.

Comparison of interviewee preferences and matches based on same-state and medical school and program affiliation were completed with base R functions.

A general linear model was created with the dependent variable of matchRec as a binary condition of whether the interview resulted in a match or not, using same-state as the geographic variable.

All further analysis and graphing were completed in R with visualizations from ggplot2.

B. Interviews per matched resident by specialty

C. Summary of continuous controls in logistic regression

**Table 4:** Summary of continuous controls in logistic regression

	25^th ^%	Mean	Median	75^th^ %	Standard Dev
Difference of USMLE score from program mean	-11.64	-3.16	-2.80	5.20	12.46
Difference of USNWR ranking from program mean	-21.38	3.37	5.31	29.27	34.89
Interviewee interview count	4	6.97	6.00	9.00	3.86
Program Doximity score	2.94	30.87	6.26	13.68	90.38

D. Frequency table of interviews by region and AOA category

**Table 5:** Frequency table of interviews by region

Region	Freq	Percent of Interviews
Mid-West	6429	23.9%
North East	7538	28.0%
South	6090	22.6%
West	6845	25.4%

**Table 6:** Frequency table of interviews by AOA category False-False (FF) = program is less than half AOA residents and the interviewee is not AOA False-False (FT) = program is less than half AOA residents and the interviewee is AOA False-False (TF) = program is more than half AOA residents and the interviewee is not AOA False-False (TT) = program is more than half AOA residents and the interviewee is AOA The value of this table is that the AOA category’s accuracy of predicting match is 35.2%, where 35.2% = (4492 + 4475)/ (4492 + 265 + 16,228 + 4,475). The 35.2% was much less than the 85.9% accuracy of the same state reported in the Results.

AOA Category (program median-candidate)	Freq	Percent of Interviews
False-False (FF)	4492	17.6%
False-False (FT)	265	1.0%
False-False (TF)	16,228	63.7%
False-False (TT)	4,475	17.6%
